# Prevalence and Underreporting of Crowned Dens Syndrome-Associated Calcifications on Cervical Spine CT in Patients with Neck Pain

**DOI:** 10.3390/jcm14248954

**Published:** 2025-12-18

**Authors:** Shira Dor, Iris Eshed, Merav Lidar

**Affiliations:** 1 Internal Medicine Department “C”, Sheba Medical Center, Tel HaShomer, Ramat Gan 5265601, Israel; shira.dor@sheba.health.gov.il; 2 Gray Faculty of Medical & Health Sciences, Tel Aviv University, Tel Aviv 6997801, Israel; iris.eshed@sheba.health.gov.il; 3 Radiology Department, Sheba Medical Center, Tel HaShomer, Ramat Gan 5265601, Israel; 4 Rheumatology Unit, Sheba Medical Center, Tel HaShomer, Ramat Gan 5265601, Israel

**Keywords:** crowned dens syndrome, calcium pyrophosphate deposition disease, neck pain, CT imaging, calcification

## Abstract

**Background:** Crowned dens syndrome (CDS) is characterized by acute neck pain and restricted motion due to calcium pyrophosphate (CPP) crystal deposition around the atlantoaxial joint. Although recognized as sufficient for the diagnosis of CPP deposition disease (CPPD), its prevalence remains uncertain. Given the high prevalence of CPPD in the general population, CDS may be more common than currently appreciated among patients with neck pain undergoing cervical spine imaging. **Methods:** This retrospective study included patients aged ≥40 years who underwent cervical spine CT for evaluation of neck pain between 2022 and 2024. Of 500 consecutive scans, 195 were eligible after excluding trauma-related, post-operative, and metastatic cases. **Results:** Periodontoid calcifications were identified in 29.2% of patients (mean age 61.5 ± 11.7 years; 37.4% male). Prevalence increased significantly with age (*p* < 0.001), reaching nearly 50% in those over 70 years. Linear calcifications were rare before 60 years (1.2%) but present in 24.5% of patients over 70. Calcifications were mentioned in only 3.5% of radiology reports. **Conclusions:** Periodontoid calcifications are relatively common in patients with neck pain, affecting nearly one-third of individuals over 40 and almost half of those over 70. Their frequent underreporting highlights a critical gap in recognition. Greater awareness and systematic reporting are warranted, as CDS may represent a common, underdiagnosed, and treatable cause of neck pain.

## 1. Introduction

Calcium pyrophosphate deposition disease (CPPD) is a prevalent form of arthritis mainly affecting individuals older than 60 years of age [1,2]. The pathogenesis of CPPD is complex and centers on the accumulation of inorganic pyrophosphate (PPi). PPi levels within the joint are regulated by enzymes such as the ecto-enzyme ENPP1 (ectonucleotide pyrophosphatase/phosphodiesterase 1). An imbalance in this regulatory system promotes CPP crystal formation, which in turn initiates an inflammatory cascade. This response is mediated through several key cellular pathways, most notably activation of the NLRP3 inflammasome, leading to the release of interleukin-1β (IL-1β) and the onset of acute symptoms such as pain, swelling, and joint dysfunction. Recent work indicates that bone abnormalities are also contributors to disease pathogenesis [2,3]. Moreover, genetic studies of rare, familial forms have identified mutations in genes such as ANKH and TNFRSF11B, suggesting unique biomolecular pathways involved in crystal deposition [2,4].

CPPD is an umbrella term encompassing three main phenotypes: acute crystal arthritis, chronic inflammatory arthritis, and an osteoarthritis-like form whose varied presentations often mimic those of other rheumatic diseases. CPPD typically involves mono-to polyarticular disease primarily affecting the knees and wrists. The chronic inflammatory form is often misdiagnosed as seronegative rheumatoid arthritis, while the pathognomonic crowned dens syndrome (CDS) represents a severe axial manifestation [4]. The most common peripheral sites of involvement are the menisci of the knee and the triangular fibrocartilage of the wrist. Yet, CPP crystals can also deposit in periarticular soft tissues, ligaments, and tendons with a predilection for the Achilles tendon and plantar fascia [5,6]. Secondary forms of CPPD are associated with underlying metabolic disorders in approximately 20% of chondrocalcinosis cases, including primary and secondary hyperparathyroidism, hemochromatosis, hypomagnesemia, and hypophosphatasia. These conditions, along with acquired risk factors such as previous joint surgery, obesity, and hypertension, are crucial contributors to the development of crystal deposition [7].

Diagnosis is based on a compatible clinical presentation and either the identification of CPP crystals in synovial fluid analysis by polarized light microscopy or biopsy, or on imaging, especially ultrasonography, particularly in patients with atypical presentations or when joint aspiration is not feasible [8].

Although synovial fluid analysis is considered the gold standard, it has high false-negative rates; therefore, diagnosis increasingly relies on imaging modalities. Conventional radiography has limited sensitivity, while ultrasound offers high sensitivity in peripheral joints. Computed Tomography (CT), which is particularly valuable for diagnosing axial involvement, and advanced techniques such as dual-energy CT (DECT) for crystal differentiation, have become increasingly important for accurate CPPD detection and classification [7].

Axial involvement of CPPD typically affects the cervical and lumbar spinal segments. CDS, characterized by calcification around the odontoid process, most prominently within the transverse ligament of the atlas, is a notable manifestation of cervical spine involvement in CPPD [7,9] and constitutes a sufficient criterion for its classification [8]. This condition presents with severe neck pain, stiffness in the neck and shoulder girdle, and occasionally with fever [10,11]. Although the presentation is often acute, chronic cases have also been described [4]. CT is considered the gold standard diagnostic modality [7,8,12].

CDS can mimic conditions such as polymyalgia rheumatica, giant cell arteritis, and, less commonly, meningitis, cervical discitis, or inflammatory spondyloarthritis [11,13]. Accurate diagnosis is essential, as CDS typically responds well to treatment with NSAIDs, colchicine, and corticosteroids [13,14], as well as IL-1 inhibitors in refractory cases [15,16].

CDS is predominantly reported in the literature through case reports and small case series, contributing to an incomplete understanding of its actual prevalence, yet emerging data suggest that it may be underrecognized and more prevalent than previously thought [17,18]. A recent meta-analysis demonstrated that CDS occurs as the sole manifestation of CPPD in approximately 85% of cases, independent of peripheral joint involvement [11]. This diagnostic difficulty is further reflected in the clinical data of the meta-analysis, where only half of the patients had an initial correct diagnosis of CDS, highlighting the frequent under-recognition of this specific axial entity [11]. Since no single clinical sign or symptom is sufficient for diagnosing CDS, reliance on imaging is essential. Among the available modalities, CT is the most accurate, rapid, and cost-effective, making it the preferred method for confirming the diagnosis [7,11].

These data support the recognition of CDS as a distinct clinical manifestation of CPPD and underscore the importance of considering it in the differential diagnosis of neck pain, a symptom with a reported prevalence of 10.4–21.3% in the general population [19].

We hypothesized that periodontoid calcifications characteristic of CDS are underrecognized and underreported despite representing a plausible cause of cervical pain. Therefore, we undertook this study to evaluate their prevalence on sequential cervical CT examinations of patients with cervical pain. Early recognition of potential CDS on cervical CT may prevent unnecessary investigations and guide appropriate anti-inflammatory treatment.

## 2. Materials and Methods

### 2.1. Study Population

This retrospective, single-center study was conducted at the Sheba Medical Center in Israel. The institution’s picture computerized archive system (PACS) was searched for cervical spine CT examinations of patients aged 40 years and older with neck pain performed between 2022 and 2023. A cohort of 500 consecutive scans was chronologically retrieved, of which 195 met the inclusion criteria. The inclusion criteria comprised patients aged 40 years and older who underwent CT for evaluation of non-specific neck pain. We excluded patients who underwent CT due to trauma-related events, metastatic disease to the spine, or for post-operative follow-up purposes. Also excluded were contrast-enhanced CT examinations since the presence of contrast material might interfere with the detection of periodontoid calcifications. Demographic and medical data were extracted from the hospital’s electronic records.

Institutional review board approval was given for the retrospective analysis of cervical CT examinations of 500 patients with neck pain. Patient consent was waived due to the retrospective nature of the study.

#### CT Examination and Evaluation

All CT examinations were carried out on two 64-slice CT scanners (ICT 956, Brilliance, Philips Medical Systems, Eindhoven, The Netherlands; VCT LightSpeed, GE Healthcare, Milwaukee, WI, USA). The slice thickness was 0.6 mm. Images were reconstructed using bone and soft tissue algorithms, reformatted into three planes, and evaluated in bone and soft tissue windows.

The presence of periodontoid calcifications was evaluated through qualitative visual assessment and classified as absent, punctate, or linear. In addition, we binarically scored the presence or absence of degenerative changes in the cervical spine.

To ensure that the prevalence data was derived strictly from objective radiographic findings, the assessment of calcifications was performed independently of the patient’s full clinical history, and the reader was provided with the patient’s age, gender, and the indication of neck pain. The CT examinations were retrospectively evaluated by one reader (a resident in internal medicine). Three introductory reading sessions with the primary reader and an experienced musculoskeletal radiologist were performed in order to develop an understanding of the different periodontoid features to be evaluated. The first 30 scans of the cohort were reviewed jointly to establish consensus on classification criteria. During the subsequent assessment of the full cohort, any borderline or uncertain findings identified by the primary reader were flagged and adjudicated with the experienced radiologist. Notably, the calcifications studied were distinct and easily recognizable as objective imaging findings, even by a non-radiologist physician who had received this structured, comprehensive training. Ten percent of the scans were also assessed by a second reader (an experienced musculoskeletal radiologist with 20 years’ experience) for reliability evaluation. Both readers were aware of the subject’s age and gender.

### 2.2. Data Analysis

Data are described as proportions for categorical variables and as means and medians for continuous variables. A Chi-square test (χ^2^) or Fisher’s exact test was used to examine the association between two categorical variables. Comparisons of continuous variables were performed using the Mann–Whitney test. Multivariate logistic regression analyses were performed to identify independent predictors, including age and sex, for the presence of periodontoid calcifications. Intraclass correlation coefficients (ICCs) were calculated for interobserver reliability by the two-way random ANOVA for absolute agreement. ICC values were interpreted as follows: 0–0.2 = poor agreement, 0.3–0.4 = fair agreement, 0.5–0.6 = moderate agreement, 0.7–0.8 = strong agreement, and >0.8 = almost perfect agreement.

Statistical analyses were performed using IBM SPSS Statistics for Windows, version 29 (IBM Corp., Armonk, NY, USA). A two-sided *p*-value < 0.05 was considered statistically significant.

## 3. Results

A total of 500 patient records of individuals who underwent cervical spine CT scans due to neck pain during the study period were reviewed. Excluded were patients whose indication for the examination was post-trauma (n = 78), metastatic disease (n = 30), post-operative follow-up (n = 122), or absence of cervical pain (n = 75). Thus, 195 patients formed the final cohort, comprising 73 males (37.4%) and 122 females (62.6%). The mean age was 61.5, the standard deviation was 11.7, and the median age was 61.

**Figure 1:**
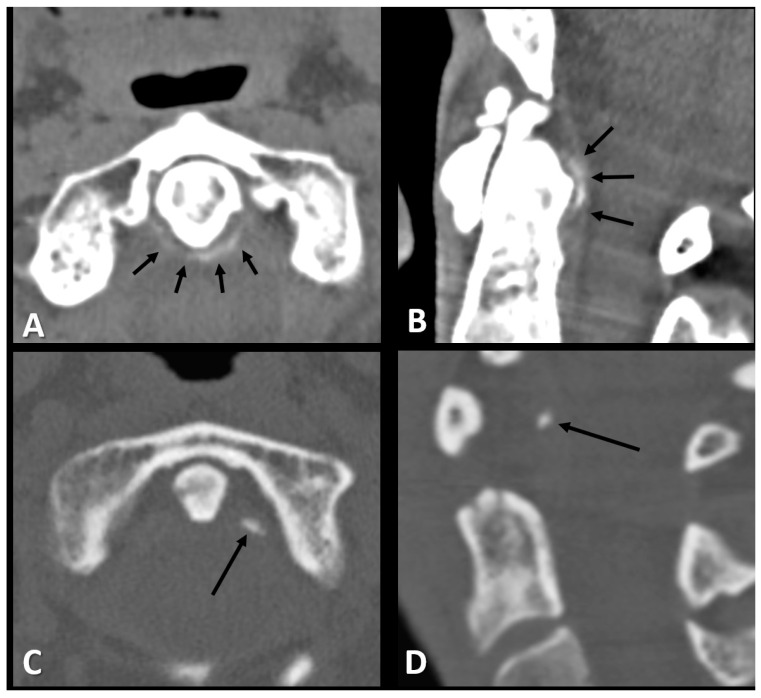
Axial and sagittal CT images of the periodontoid area of a 72-year-old woman (**A**,**B**) and a 52-year-old woman (**C**,**D**) with neck pain demonstrating the two types of periodontoid calcifications, linear (short arrows in (**A**,**B**)) and punctate (long arrow in (**C**,**D**)).

Among the study population, 57 patients (29.2%) had periodontoid calcifications (linear: 23 patients, 39.6%, punctate: 34 patients, 59.6%). Representative examples of the two calcification patterns observed in the study cohort are presented in Figure 1. Periodontoid calcifications were explicitly mentioned in only two of the positive radiology reports (3.5%), whereas in the remaining 55 reports (96.5%), they were not referenced.

**Figure 2:**
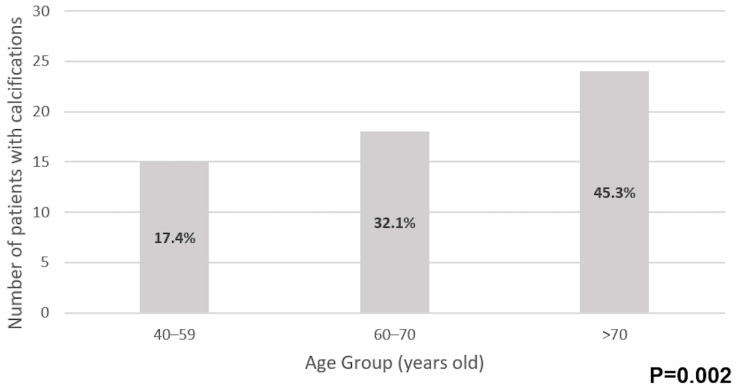
Prevalence of periodontoid calcifications by age group.

The prevalence of periodontoid calcifications significantly increased with age, with 17.4% of patients aged 40–59 years (15/86) having calcifications, compared to 32.1% in the 60–70 years’ age group (18/56) and 45.3% in those above 70 years (24/53), *p* = 0.002 (Figure 2).

**Figure 3:**
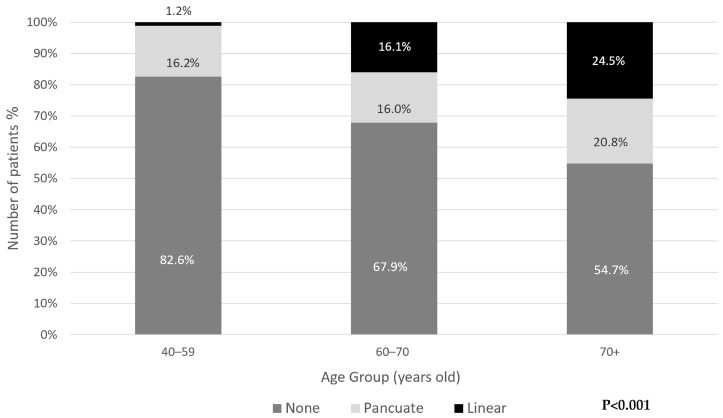
Distribution of periodontoid calcifications type by age group.

There was a significant association between age and the type of calcification. Generally, punctate calcifications were the most common in all age groups. Younger individuals (40–59) predominantly had punctate calcifications (98% of all calcifications in this age group), whereas linear calcifications were more prevalent in older age groups, reaching 24.5% of those over 70 years old (*p* < 0.001) (Figure 3).

A higher proportion of periodontoid calcifications was observed in males, with 36.9% of males (27/73) and 24.6% of females (30/122) having calcifications, but the difference is not statistically significant (*p* = 0.065). There was no significant association between sex and the type of calcification (*p* = 0.09).

Multinomial logistic regression revealed that age was significantly associated with linear calcifications, with older individuals having a higher likelihood of developing linear calcifications compared to those with no calcifications (OR = 0.032, 95% CI: 0.004–0.279, *p* = 0.002). The regression analysis was adjusted for sex and the degree of odontoid osteoarthritis, neither of which showed a statistically significant association with any type of calcification (*p* > 0.05).

Cervical degenerative changes were significantly associated with the presence of periodontoid calcifications, even after adjustment for age.

Three of 195 scans (1.5%) demonstrated osteoporotic fractures that could likely account for the patients’ symptoms. In the remaining cases, aside from degenerative changes and periodontoid calcifications, no additional findings were observed.

Interobserver reliability for CT periodontoid calcifications assessment was 0.973, indicating almost perfect agreement between the two independent observers.

## 4. Discussion

The present study demonstrated that periodontoid calcifications were present in 29.2% of cervical spine CT scans performed for neck pain. Prevalence increased with age, with nearly half of patients over 70 years affected. Notably, linear calcifications were rare before 60 years but observed in almost one-quarter of patients above 70, indicating an age-dependent increase in both the frequency and extent of calcification.

Our results expand on prior work. Sano et al. reported a lower prevalence (15.9%) in patients undergoing brain CT regardless of clinical presentation [20]. This difference in prevalence could be attributed to the inclusion criteria in our study. We aimed to evaluate a symptomatic population, and our cohort consisted of patients who underwent cervical CT due to clinical neck pain. Whereas Sano et al.’s cohort primarily included asymptomatic individuals who underwent brain CT for unrelated indications.

Nevertheless, both studies observed an age-dependent rise in prevalence, and Sano et al. also found that 12.5% of those with calcifications developed CDS [20]. Similarly, Kobayashi et al. identified calcifications in 63% of patients presenting with neck pain compared with 13.5% of asymptomatic controls [21], reinforcing the link between calcification and clinical symptomatology. Our findings, particularly the high prevalence in elderly individuals, align with these observations and support the notion that periodontoid calcifications are an important, and likely underrecognized, contributor to neck pain. This finding aligns with a small study by Roverano et al. [12], who evaluated the prevalence of periodontoid calcifications in patients with established articular chondrocalcinosis and compared the results with a control group. They reported a markedly elevated prevalence of 71% in the study cohort, whereas no calcifications were identified in the control group (*p* < 0.0001). Furthermore, among patients with detectable calcification, 45% exhibited clinical features consistent with CDS [12]. Importantly, we observed that calcifications were present in a substantial proportion of patients aged 40 years and older, suggesting that CDS should not be regarded solely as a disease of the elderly. Even when deposits were limited to punctate or early linear forms, they may still represent a clinically relevant cause of neck pain in middle-aged adults. This observation broadens the potential spectrum of patients at risk and suggests that CDS may be more prevalent in younger populations than previously recognized.

Morphologically, punctate calcifications predominated in younger patients, whereas linear calcifications were strongly associated with advancing age. This novel observation, where linear calcifications were rare in younger patients yet reached 24.5% in patients above 70 years, highlights a significant age-related difference in calcification pattern. While further longitudinal investigation is warranted, this association suggests that the linear pattern may represent a long-standing crystal deposition burden. Previous reports have described punctate and linear forms of calcification in calcium pyrophosphate deposition (CPPD) disease [21,22,23], but, to our knowledge, no prior study has detailed their age-related distribution.

Despite their frequency, periodontoid calcifications were infrequently reported in radiology interpretations. This underreporting may be due to limited awareness of their clinical relevance and the lack of standardized reporting guidelines. Periodontoid calcifications are not routinely the primary focus of cervical CT interpretation unless specifically requested by the referring clinician. In addition, awareness of crowned dens syndrome among both clinicians and radiologists may be limited, which could contribute to missed documentation of these findings.

Given the 2023 ACR/EULAR classification criteria, which allow for a diagnosis of CDS on imaging alone in the absence of alternative explanations [8], under-recognition may contribute to diagnostic delays or misdiagnosis. We suggest focusing future efforts on clinical awareness and reporting protocols. Given that a CT scan is already a highly sensitive modality for detecting these calcifications, the primary emphasis should be placed on raising awareness among radiologists and clinicians about the significance of periodontoid calcification. Encouraging a systematic approach to evaluating and documenting these findings in cervical spine CT scans performed for neck pain may help address the current gap between radiological observation and clinical diagnosis, ultimately promoting timely and appropriate patient management. This gap is also reflected on the clinical side, as imaging referrals frequently fail to explicitly mention suspicion of CDS as part of the differential diagnosis.

Although clinical and laboratory data necessary for a definitive diagnosis of CDS were not available, the uniform presentation of neck pain across our cohort, combined with the radiological findings, suggests that some individuals with calcifications may have had unrecognized CDS. Furthermore, the increasing burden of linear calcifications in elderly patients may parallel the well-documented association of CPPD with greater flare frequency and severity in older populations [11,24].

This study has several limitations. Its retrospective design limited the ability to evaluate symptom onset, duration, and severity, and we lacked information on comorbidities, medication use, or prior history of CPPD. In addition, the absence of follow-up data precluded assessment of long-term outcomes or treatment response. The primary evaluation was performed by a non-radiologist physician, following focused introductory training and with close supervision by a senior musculoskeletal radiologist, and the resulting ICC demonstrated almost perfect agreement. The assessment in this study relied on qualitative visual interpretation without quantitative confirmation. Although the evaluated finding is relatively distinct and readily recognized on CT, this should be acknowledged as a methodological limitation.

Nonetheless, strengths include the relatively large cohort, systematic imaging evaluation, and novel assessment of age-related morphological patterns of calcification.

Future research should prioritize large-scale studies with comprehensive clinical and laboratory data, thereby overcoming the current data limitations of this study. Such studies are necessary to better clarify the natural history of periodontoid calcifications, identify broader risk factors for their development, and specifically evaluate the prognostic significance of the observed morphological patterns (punctate versus linear) on long-term clinical outcomes. Furthermore, given the critical need to close the diagnostic gap demonstrated in this study, prospective interventional studies assessing the direct impact of newly implemented systematic reporting protocols on both the detection rate of CDS and subsequent patient management would be valuable.

## 5. Conclusions

Periodontoid calcifications were identified in nearly one-third of patients presenting with neck pain, affecting close to half of those aged over 70 years and a substantial proportion of those aged 40 years and older. These findings indicate that crowned dens syndrome may represent a common and underrecognized cause of neck pain, not only in the elderly but also in middle-aged populations. Despite their frequency, calcifications were rarely reported in radiology interpretations, underscoring a gap in clinical awareness and radiological reporting. Given their characteristic imaging appearance, diagnostic value under the 2023 ACR/EULAR criteria, and responsiveness to treatment, periodontoid calcifications should be routinely considered in the differential diagnosis of neck pain. Greater recognition and systematic reporting have the potential to improve diagnostic accuracy, avoid unnecessary investigations, and facilitate timely and appropriate management.

## Data Availability

The data presented in this study are available upon reasonable request from the corresponding author. The data are not publicly available due to the sensitive nature of the patient data and ethical restrictions related to preserving participant confidentiality, which prohibit the public sharing of raw clinical data.
